# The Effect of Chinese Traditional Medicine Huaiqihuang (HQH) on the Protection of Nephropathy

**DOI:** 10.1155/2020/2153912

**Published:** 2020-06-16

**Authors:** Xueyan Zhang, Yiyu Cheng, Qian Zhou, Haojie Huang, Yinmiao Dong, Yang Yang, Mingyi Zhao, Qingnan He

**Affiliations:** Department of Pediatrics, The Third Xiangya Hospital, Central South University, Changsha, Hunan Province, China 410013

## Abstract

Kidney disease is one of the common diseases with high morbidity and high mortality, which brings a huge burden to the society and the patient's family. The pathogenesis, treatment, and prognosis of kidney diseases are related to oxidative stress, inflammation, mitochondrial damage, and immune dysfunction. However, existing treatments always cause some damage to the kidneys. Kidney disease and immunosuppressant used together often lead to drug toxicity, patients with weakened immunity, organic rupture of the normal structure of the kidney, damage to the physiological function of the kidney, etc. Huaiqihuang is a kind of traditional Chinese medicine with a history of more than one thousand years. According to research, *Robinia pseudoacacia* can regulate the immune function by regulating oxidative stress, calcium inflow, and mitochondrial ATP. At the same time, it is also involved in regulating the ways of cell death, such as apoptosis, autophagy, ferroptosis, pyroptosis, and clockophagy, to reduce kidney damage, which has important clinical value. This article reviews the exact mechanism and clinical application of Huaiqihuang in different types of nephropathy. The aim is to provide new ideas for the treatment of clinical nephropathy.

## 1. Introduction

Due to the unhealthy lifestyles, drug abuse, birth defects, infections, and other phenomena in modern people, the incidence of kidney disease is also increasing globally, which has caused widespread concern in the society. Various nephropathies, such as glomerulonephritis, renal tubular injury, and renal vascular disease, as well as some common complications, such as hypertension and diabetes, are common clinical kidney system diseases [1]. Its disability rate, death rate, and growth rate remain the top of all the chronic diseases. According to the existing studies, a large number of kidney diseases are caused by immune factors, such as membranous nephropathy, and mesangial proliferative glomerulonephritis [2]. At present, most of the clinical treatments are symptomatic treatment, with poor efficacy and prognosis, and sometimes, it even causes greater side effects. Such long-term treatment is a serious financial burden for many families. Therefore, some novel treatments need to be explored and applied. Although the specific pathogenesis of many diseases is still unclear, some studies have shown that they are often related to oxidative stress, stimulating autophagy, and immune dysfunction [3–5].

Currently, new findings imply that Huaiqihuang (HQH), a mixture of Chinese herbs, is mainly composed of *Trametes*, *Lycium barbarum*, and *Polygonatum*; all of which have been used extensively in China for thousands of years and found to be effective in treating kidney disease [6, 7]. It not only enhances immune function and improves curing effectiveness but also reduces toxicity of medicine [8].

It has been revealed that HQH has a therapeutic effect for kidney diseases, such as primary nephrotic syndrome (PNS), IgA nephropathy (IgAN), and mesangioproliferative glomerulonephritis (MsPGN) [9]. Mounting evidence demonstrated a directly protective link between HQH and renal intrinsic cells. HQH can protect renal tubular epithelial cells by downregulating the levels of E-cadherin transcriptional repressors ZEB1, ZEB2, and *α*-SMA, subsequently slowing down the epithelial-mesenchymal transition [10]. It also has the ability to inhibit inflammatory cytokine expression like IL-6 and macrophage infiltration [11]. The oxidative stress levels are suppressed with decreased Ca^2+^ and ROS expression in podocyte [12]. By means of autophagy, HQH balances the activity between mTOR and HQH [12, 13]. HQH can also inhibit the apoptotic p-ERK/CHOP signaling pathway and regulate the Bcl-2/Bax signaling pathway to protect renal intrinsic cells [12]. Li et al. reported that Huaiqihuang can effectively reduce proteinuria in patients with IgAN and significantly reduce hematuria [14]. Zhu et al. found that HQH reduces podocyte injury and albuminuria by inhibiting the expression of inflammatory cytokine ADRN in rats [11]. Moreover, Pan et al. demonstrated that HQH has a broad effectiveness in the treatment of immunological diseases and cancers [6]. Some pharmacological studies showed that HQH is a biological response regulator, which may either enhance immunity or have effects like anti-inflammatory, antioxidant, and anticancer. The evaluations of serum renal function parameters have proved HQH almost has no cytotoxicity to normal kidney. All the researches show that HQH is likely to become an adjuvant drug for kidney diseases [11, 12, 15].

**Figure 1:**
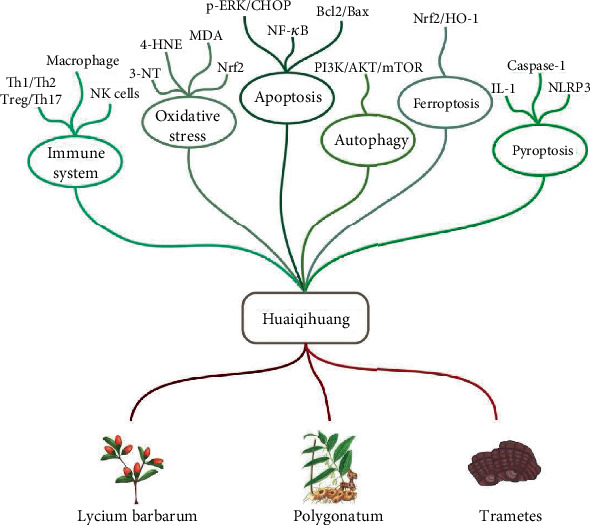
Specific components and mechanisms of HQH.

This article reviews up-to-date researches on the function of HQH and relative treatment for renal diseases, providing a theoretical basis and treatment strategy for further clinical application. The specific components and mechanisms of HQH can be summarized in Figure 1.

## 2. Mechanism

### 2.1. Regulating the Function of the Immune System

The characterization of CD4+ T-cells reflects the immune status and is important in the maintenance of tumorigenesis and homeostasis. HQH markedly increased the classic Th1 cytokine IFN-*γ* and the Treg cytokine IL-10 and TGF-*β* but decreased the Th2 cytokines IL-4, IL-5, and IL-13 and the Th17 cytokine IL-17, indicating that it regulates both the Th1/Th2 balance and the Th17/Treg balance [16]. Li et al. found that patients with renal cell carcinoma (RCC) are observed to have a skewed in Th1/Th2 and Th17/Treg balance [17]. He et al. also proved that the imbalance of Th1/Th2 proinflammatory cytokines plays an important role in the development and progression of IgA nephropathy (IgAN), suggesting that IgAN is associated with the upregulation of Th2 lymphocytes [18]. Tsuruga et al. also found a possible role for an imbalance in Th1 and Th2 proinflammatory cytokines in the development and progression of glomerulonephritis [19]. The above evidence proves that the imbalance of Th1/Th2 and Th17/Treg, to some degree, contributes to kidney diseases [19]. In the experiment of mice, Liang et al. found that HQH could regulate Th1/Th2 and Treg/Th17 via the rebalance of cytokine profiles and change the mRNA expression levels of the transcription factors [16]. The levels of IFN-*γ* in plasma were positively correlated with the dosage of HQH [20]. Interestingly, distortion of the Th17/Treg balance favoring the proinflammatory Th17 side is suspected to contribute to exacerbation of autoimmune disorders [18, 21].

Macrophages are vital sources of proinflammatory cytokines and injurious mediators in various types of acute kidney diseases [2]. Generally, they are classified into classically activated macrophages (M1) or alternatively activated macrophages (M2), depending on cell markers and cytokines produced, such as CD16, CD32, TNF, IL-6, Arg1, and CD206 [22]. HQH may play a certain anticancer effect in some kinds of kidney cancer by polarizing macrophages from M2 to M1 to enhance phagocytosis and inhibiting the angiogenesis of tumor tissue. The M2 phenotype helps in forming the tumor microenvironment (TEM) and thus becoming an influence factor of some kidney cancers [6]. Li et al. found that Huaier suppressed cell motility and reduces M2 markers, including CD206, Mrc-2, Arg-1, and IL-10. The number of M2 in tumor was declined after Huaier treatment, but the NO production and the M1 marker iNOS were increased. Also, HQH can inhibit and even reverse the polarization from M1 to M2 both quantitatively and functionally, thus playing a significant role in antagonizing tumor progression.

What is more, tumours tend to grow when the body's NK cells are less active [23]. Pan et al. found that Huaier can enhance NK cell activity and play an antitumor role [6]. Using Huaier in rats with hepatocellular carcinoma (HCC) increases the number of NK cells, which has a certain therapeutic effect on liver cancer [24]. With the treatment for NS patients with both HQH and hormones, NK cells increase in patients and it was more effective than that of patients treated with hormone alone.

### 2.2. Regulation of Oxidative Stress

When stimulated, a large number of free radicals such as reactive oxygen species (ROS) and reactive nitrogen species (RNS) will be produced, which breaks the balance between the oxidation mechanism and the antioxidant mechanism and leads to oxidative stress. Oxidative stress is a vital part in kidney injury [25]. ROS can activate the mitogen-activated protein kinase (MAPK) signaling pathway and lead to renal tubular cell death. Subsequently, ROS promotes the fibrotic process by enhancing inflammation. Fibrosis and inflammation themselves may in turn generate an increase in ROS formation [3]. Oxidative stress markers such as 4-HNE, 3-NT, and malondialdehyde (MDA) can evaluate the progression of kidney disease, suggesting that kidney damage is related to oxidative stress [26]. Shopit et al. found that activation of the Nrf2/ho-1 pathway can protect kidney cells from oxidative stress [27]. Fang et al. found in a cisplatin nephrotoxic rat model that activation of the Nrf2/HO-1 pathway significantly reduced intracellular ROS and decreased the expression of oxidative stress markers [8].

According to the existing experimental results, Chinese medicine HQH can alleviate the oxidative stress damage of cells and has a certain therapeutic effect on kidney diseases. Duan et al. found that in patients diagnosed with primary glomerular disease, malondialdehyde (MDA) levels were significantly lower after the administration of HQH than that of the control group [26]. HQH is also valuable in the treatment of podocyte dysfunction caused by hyperglycemia. When MPC5 podocytes were cultured in a high glucose medium, intracellular Ca2+ and ROS levels increased significantly, leading to mitochondrial dysfunction. Li et al. [12] showed that Huaier can improve the mitochondrial membrane potential of podocytes, restore the level of Ca2+ in the cytoplasm, and inhibit the production of ROS, thereby reducing the level of oxidative stress. These studies suggest that HQH can enhance the antioxidant activity of the kidney, so as to play a certain protective role.

### 2.3. Inhibiting the Apoptosis

Apoptosis is a process of programmed cell death, characterized by cell volume reduction, chromatin condensation, DNA internucleosome lysis, and apoptotic body formation [28]. Apoptotic cells are rapidly engulfed by macrophages, preventing the release of intracellular components and inflammatory factors, leading to the noninflammatory cell death. Abnormal apoptosis can cause kidney diseases such as polycystic kidney disease (PKD) [29]. HQH can reduce cell apoptosis and alleviate renal injury [29–31]. The specific mechanisms are depicted as follows.

#### 2.3.1. Regulating the P-ERK/CHOP Pathway

Endoplasmic reticulum stress (ERS) is a major cause of apoptosis [32]. Extracellular regulatory protein kinase (ERK) is a member of the MAPK family. McCullough et al. found that ERS can activate the p-ERK/CHOP pathway and reduce the expression of antiapoptotic protein Bcl-2 [33], thus promoting apoptosis and damage to podocytes [31, 34]. Li et al. cultured rat MPC5 podocytes to establish the ERS model and found that p-ERK and CHOP expressions increased after podocyte injury [35].

HQH can protect podocytes by inhibiting the p-ERK/CHOP signaling pathway and inhibiting cell apoptosis. HQH also has therapeutic effect on patients with proteinuria [36]. HQH also significantly reversed the hg-induced upregulation of glucose-related protein 78 (GRP78) and alleviated the dysfunction such as podocyte apoptosis and DNA damage. As an indicator of endoplasmic reticulum stress, C/ebph protein was also changed [12]. In summary, HQH inhibits cell apoptosis by inhibiting endoplasmic reticulum stress and has a therapeutic effect on podocyte dysfunction-related renal diseases.

#### 2.3.2. Regulating the Bcl-2/Bax Pathway

The homeostasis of the Bcl-2 to Bax ratio plays an important role in endogenous apoptotic pathways [30]. After the apoptotic signal is issued, the expression of proapoptotic protein Bax in the cytoplasm promotes the release of cytochrome C (Cyt C) from the mitochondria into the cytoplasm, forms the apoptotic complex, initiates caspase cascade, and leads to cell apoptosis [36].

According to Liu et al., HQH was found to downregulate the expression of proapoptotic proteins Bax, Cyt C, and caspase 3 and upregulate the expression of antiapoptotic proteins Bcl-2 [9]. Similarly, Fang et al. found that, compared with the control group, the proportion of proapoptotic and antiapoptotic proteins in the HP-1 treatment group has a relatively balanced ratio and the apoptosis was reduced accordingly [8, 37]. Furthermore, GRP78 can bind and inhibit the activity of proapoptotic protein BIK on endoplasmic reticulum [38], promoting the expression of bcl-2 so as to inhibit cell apoptosis [39, 40]. Li et al. treated high-glycemic MPC5 podocytes with Huaier and found that they could significantly reverse GRP78 upregulation and regulation of the Bcl-2/Bax signaling pathway. It can also inhibit the expression of caspase 3 and reduce podocyte damage [12].

#### 2.3.3. Regulating the NF-*κ*B Signaling Pathway

RelA (p65), RelB, c-Rel, p50, and p52 are transcription factors in the NF-*κ*B family. Inhibitors of nuclear factor regulate transcription of the NF-*κ*B pathway, thereby regulating apoptosis [30]. Liu et al. demonstrated that Huaier inhibits the expression of TNF-*α*, p-NF-*κ*B p65, and I*κ*B, thereby inhibiting the apoptotic NF-*κ*B signaling pathway and protecting the kidney [9]. Guo et al. found that in CP-induced nephropathy, Huaier can reduce the TLR4/NF-*κ*B pathway to protect the kidneys [37]. Adding Huaier to CP-induced nephropathy, it was found that it can downregulate the PI3K/Akt/mTOR/NF-*κ*B signaling pathway in cisplatin nephrotoxicity cells and downregulate the expression of p-NF-*κ*B. These can never emphasize more that HQH can reduce apoptosis and promote the survival of kidney cell through the NF-*κ*B signaling pathway [8].

### 2.4. Regulated Autophagy

Autophagy is a dynamic cell balance mechanism of energy and resources, which plays a role through the degradation and circulation of lysosomes in cells [4]. This in turn helps cells maintain their integrity more effectively by regenerating metabolic precursors and removing subcellular debris [41]. Autophagy activation and inhibition have been associated with acute kidney injury, chronic kidney disease, diabetic nephropathy, and polycystic kidney disease in clinical studies. After acute kidney injury, autophagy can protect renal tubules from apoptosis and promote cell regeneration. However, abnormal autophagy can lead to loss of podocytes, injury of proximal tubular cells, and glomerulosclerosis [4, 42, 43]. Autophagy also plays an important role in the physiological functions of podocytes [13] and excessive reduction or increase of mTOR activity can cause podocyte damage [44, 45]. For example, in the diabetic nephropathy (DN) model, the activated PI3K/Akt/mTOR pathway can reduce the autophagy level of podocytes, leading to podocyte damage [46]. Cybulsky et al. believed that in diabetic nephropathy, podocyte autophagy was impaired, and the recovery of autophagy attenuated podocyte injury [42].

Fang et al. found that activation of autophagy can cause cp-induced oxidative damage [8]. Huaier can reduce autophagy by regulating the mTOR/PI3K/Akt pathway and inhibit the expression of autophagy-related proteins such as Beclin1 and LC3-II. It also promotes the recovery of mitochondrial function, suggesting that Huaier can alleviate nephrotoxicity [36]. In addition, Fu et al. confirmed that the occurrence of renal cell carcinoma was related to the activation of the PI3K/Akt/mTOR pathway [47]. Wei et al. found that treated with Huaier, the expression of the downstream protein p70S6K of mTOR decreased in renal cell cancer cell 786-O, indicating that Huaier has a potential inhibitory effect on renal cell carcinoma through the PI3K/AKT/mTOR/p70S6K pathway [48]. The balance between mTOR and autophagy activity of podocytes can be maintained by HQH, suggesting its potential value in protecting glomerular function and preventing related diseases [12, 35].

### 2.5. Regulating the Ferroptosis

Ferroptosis is dependent upon intracellular iron, but not other metals, and is morphologically, biochemically, and genetically distinct from apoptosis, necrosis, and autophagy, which is a form of oxidative cell death characterized by accumulation of intracellular lipid reactive oxygen species (ROS) [49]. Current studies have found that activation of the Nrf2/HO-1 signaling pathway can inhibit ferroptosis [50]. Nrf2 is a key transcription factor that resists ferroptosis. HO-1 is an important source of intracellular iron and a key enzyme to induce ferroptosis [51]. Nrf2 was relocated and entered the nucleus to increase the expression of HO-1, which ultimately reduced the oxidative damage of cells [52, 53].

Fang et al. treated kidney cells of mice with chemotherapy drug cisplatin (CP), finding that intracellular ROS increased and glutathione (GSH) decreased [8]. Reduced GSH can lead to a decreased GPX activity and attenuated antioxidant capacity [54], inducing ferroptosis easily [55]. After treatment with Huaier polysaccharide (HP-1), intracellular ROS decreased, GSH activity was restored, and thus the activating Nrf2/HO-1 signaling pathway. Thus, the combination of cisplatin and HP-1 may inhibit the occurrence of ferroptosis and improve the antioxidation of the kidney.

### 2.6. Regulating the Pyroptosis

Inflammatory activation, usually consisting of a pattern recognition receptor (PRR), an apoptosis-associated speck-like protein (ASC), and a cysteine protease (caspase-1), leads to the secretion of inflammatory cytokines, which subsequently lead to cell death. This pattern of inflammatory cell death is known as pyroptosis. The mechanism of occurrence and regulation is different from that of apoptosis, necrosis, and other ways of cell death [56, 57]. Several studies have showed that inflammasomes are closely related to kidney diseases, including the NOD- and LRR-pyrin domain-containing 3 (NLRP3) inflammasome, especially NLRP3, which play a role in regulating kidney inflammation and fibrosis [58]. The NLRP3 protein contains three different domains, namely, a central nucleotide-binding NACHT domain (NOD domain) responsible for self-oligomerization during activation, C-terminal leucine-rich repeat (LRR) which is a recognition domain for different ligands, and an N-terminal pyrin domain (PYD) mediating the interaction with proteins. According to Ke et al. the expression levels of NLRP3 and caspase-1 are significantly elevated in the kidneys of CKD or fibrosis patients and the NLRP3 inflammasome may be activated and involved in the regulation of renal fibrosis [59]. It has been previously reported that renal tubular epithelial cells can express and release IL-18, indicating that the NLRP3 inflammasome and caspase-1 are also present within renal tubular epithelial cells [60].

According to the research of Wang et al., Huaier inhibited NLRP3 inflammasome activation-induced IL-1*β* secretion and caspase-1 cleavage. Moreover, Huaier decreased NLRP3 protein expression via promoting NLRP3 degradation through the autophagy lysosome pathway. And their research findings demonstrate a novel function for Huaier in the regulation of NLRP3 inflammasome activation and suggest a potential role for Huaier in NLRP3 inflammasome-associated diseases such as renal fibrosis [61]. So far, the research on HQH-induced pyroptosis is limited and further experiments require to be verified. This may provide new directions for the treatment of clinical nephropathy in the future.

## 3. Applications

HQH is a mixture of Chinese herbal medicines. It has been widely used in China for thousands of years. In recent years, research has shown that HQH can effectively treat kidney diseases. HQH is a biological response modifier with strong and wide clinical adaptability, as well as anti-inflammatory, antioxidant, and anticancer effects. In addition, it can not only enhance the immune function but also improve the healing effect. The most important thing is to reduce the toxicity of the drug, thus reducing toxicity after treatment. Studies have shown that HQH has a therapeutic effect similar to glucocorticoid on doxorubicin-induced primary nephrotic syndrome, and the Chinese herbal compound preparation also has a good therapeutic effect on glomerulonephritis. But compared to other drugs, HQH has almost no toxicity to normal kidney cells, can long-term use, and has a role in protecting the kidneys. Therefore, HQH is very likely to be an auxiliary drug for kidney disease and put into clinical use.

### 3.1. Mesangioproliferative Glomerulonephritis (MsPGN)

The mesangioproliferative glomerulonephritis (MsPGN) is a renal disease characterized by the proliferation of mesangial cells and deposition of extracellular matrix [62], even leading to end-stage renal failure. However, there are still no clear mechanisms explaining this kidney disease, and the current treatment is still controversial without a convincing outcome during treatment [2, 10, 14]. Therefore, it is necessary to develop new drugs to improve the therapeutic effect.

Anti-Thy1 glomerulonephritis is a well-characterized experimental model that is a simulation of human MsPGN. According to the research of Bai et al., PDGF-B is involved in the development of mesangial cells and overexpression of glomerular PDGF-B has an important effect on MsPGN disease. The research indicates that HQH can reduce the stimulation of PDGF-B, decrease the urinary protein, prolong G2 phase, and inhibit cell proliferation by regulating cyclin, so as to reduce the proliferation of anti-Thy-1 MsPGN [15]. As a result, HQH inhibits mesangial proliferation in MsPGN, promising a possible therapy in its clinical treatment.

### 3.2. IgA Nephropathy

IgA nephropathy is considered an immune complex deposition disease, characterized by recurrent hematuria and proteinuria with precursor infection [8]. The pathogenesis of IgA nephropathy is unclear, but it is mainly associated with immune dysfunction and recurrent infection [14]. Considering that ESRD is still developed in lots of IgAN patients and no drug has a significant effect, a shift from the traditional therapies should be needed.

Geng et al. have found that there are decreased amount of mRNA and nephrin protein in IgA nephropathy. Effectively, HQH can increase the expression of nephrin and improve the anomalies of their distribution, therefore stabilizing the function of glomerular filtration membrane [7, 10]. Studies of Li et al. have shown that IgG is significantly higher after treatment with HQH than before treatment and CD3; CD4 are significantly higher than before treatment. Therefore, HQH can regulate the immune function and reduce infection in children with IgA nephropathy, indicating a quickly and effectively to treat it^15^. IgA can be improved by the antiproliferation and immunomodulatory properties of HQH. In addition, HQH is found to be able to effectively reduce hematuria and urinary red blood cell count [14].

Based on the fact above, HQH may play an important role in reducing mild proteinuria, hematuria, and delaying the progression of CKD in patients with primary glomerular disease, so it may be an effective and conservative therapy for mild IgA nephropathy patients, including children and adults [7, 8, 10]. However, there is no effective evidence that HQH is effective to severe patients; as a result, further studies should be required.

### 3.3. Primary Nephrotic Syndrome

Primary nephrotic syndrome (PNS) is a frequently occurring disease in the urinary system in childhood with the acceleration of modernization and urbanization, which is characterized by massive proteinuria, hypoproteinemia, hyperlipidemia, and edema [16–18]. It is caused by increased permeability of glomerular filtration membrane to plasma protein and loss of large amounts of plasma albumin from urine.

Geng et al. suggest that HQH granule can reduce the incidence of infection and disease recurrence in patients with nephrotic syndrome by reducing the inflammatory effects of IL-18 and enhancing the inflammatory inhibitory effects of IL-10. It is also reported that HQH granule can reduce the excretion of urine protein by regulating the p-ERK/CHOP signaling pathway to promote podocyte proliferation and suppress podocyte apoptosis. Data confirmed that the auxiliary treatment of HQH granules on nephrotic syndrome was safe, and no adverse reactions such as allergy, liver, and kidney function damage and bone marrow suppression were found [7]. In addition, HQH granule combined with other treating medicine has proven to be effective in children with primary nephrotic syndrome, which can effectively relieve clinical symptoms and enhance immune regulation function, and has positive clinical application and promotion value [5, 7, 15].

### 3.4. Allergic Purpura Nephritis

Allergic purpura nephritis is mainly mesangial proliferative glomerulonephritis. The proliferation of mesangial cells and excessive deposition of extracellular matrix are the pathological basis of various mesangial proliferative nephropathies. The clinical manifestations of allergic purpuric nephritis vary greatly. The mild ones only have hematuria and proteinuria. In severe cases, nephrotic syndrome can occur, and even renal function declines. Glomerular mesangial cells secrete TGF-beta 1, which regulates the rate of proliferation and synthesis of stromal proteins by binding to corresponding receptors on the mesangial cells. Therefore, inhibition of TGF-*β*1 expression is the key to treatment and even prevention of its occurrence and development.

Studies have shown that HQH particles can effectively downregulate the expression of TGF-*β*1, so as to have a good effect on allergic purpura nephritis [63, 64]. Clinical studies have shown that HQH granules can maintain the integrity of the cerebral venous filtration barrier by upregulating the expression of nephron and podocin on the septal septum, thereby reducing the leakage of urinary protein. Some researchers have pointed out that HQH granule has a good clinical effect in improving allergic purpuric nephritis in adults with 24-hour urine protein quantitation and renal function [65].

### 3.5. Adriamycin-Induced Nephropathy

Adriamycin nephrosis (AN) in rats is a representative animal model of nephrotic syndrome, pathologically characterized by extensive tubular injury, interstitial inflammation, and renal fibrosis [66]. Using this model, some studies can better study nephrotic syndrome by studying the relationship between globulin ultrafiltration and interstitial fibrosis. Zhu et al. discovered that HQH can significantly reduce proteinuria and prevent podocyte injury by inhibiting the expression of uninflammatory cytokines in ADRN rats, but its underlying mechanism remains unclear [11]. Moreover, HQH improves renal tubule interstitium by inhibiting inflammatory cytokine expression and macrophage infiltration. Liu et al. suggest that HQH can reduce adriamycin-induced apoptosis, while NF-*κ*B signaling is inhibited, and it is speculated that HQH may interfere with Bcl-2/Bax signaling pathway in other ways, thereby improving the apoptosis of ADRN rats [9]. Thus, HQH might hold promise for a clinical application involving the interference with the pro-inflammatory and pro-apoptotic signaling and the treatment of CKD.

### 3.6. Renal Interstitial Fibrosis

Renal fibrosis represents the common ultimate pathway of almost all chronic and progressive nephropathy, involving glomerular sclerosis and interstitial fibrosis. The model of unilateral ureteral obstruction (UUO) produces progressive renal fibrosis [67]. Renal interstitial fibrosis (RIF) is characterized by the expression of inflammatory cells and the accumulation of collagen components in the renal interstitial, atrophy and dilatation of renal tubules, capillary reduction around the tubules, and progressive destruction of the nephron. Renal interstitial fibrosis eventually leads to renal failure [4, 10, 37, 42, 66].

Studies of Pu et al. have shown that HQH alleviates renal fibrosis by reducing infiltration of myofibroblast and downregulating the expression of *α*-SMA. Furthermore, in recent years, research shows that low-dose HQH cream cannot significantly reduce renal interstitial damage in UUO rats and improve interstitial fibrotic lesions. And medium- and high-dose HQH cream can reduce the degree of renal interstitial fibrosis in UUO rats. In summary, HQH cream reduces renal interstitial fibrosis in a dose-dependent manner [3, 10, 11, 67].

### 3.7. Cisplatin Nephrotoxicity

Cisplatin (CP) is a commonly used chemotherapy drug for the treatment of malignant tumors. But various side effects such as nephrotoxicity, ototoxicity, and neurotoxicity, affecting its efficacy [37]. Liang et al. investigated that Huaier can reduce the levels of oxidative stress, inflammation, and mitochondrial dysfunction, thereby reducing kidney damage. Huaier can also significantly inhibit cp-induced renal tubular apoptosis and cell cycle arrest [8]. Therefore, HQH can be used to prevent the nephrotoxicity of cisplatin without affecting the antitumor effect of cisplatin, so as to protect the kidney to a certain extent. It has a great auxiliary medicinal value in clinical practice.

### 3.8. Renal Carcinoma

Renal parenchymal carcinoma is an adenocarcinoma of renal tubular epithelial cells, of which 85% is clear cell carcinoma. It grows in the renal parenchyma, infiltrates, compresses, and destroys the renal pelvis and calices, develops toward the renal capsule, and forms hemangioma plugs or metastases to lymph nodes and other organs.

According to the research of Wei et al. about the Huaier's antitumor effect on 786-O (human renal clear cell carcinoma cell), Huaier can inhibit cell growth, proliferation, migration, and invasion of renal clear cell carcinoma cell [48]. Proliferation and migration of cancer cells are key processes in the progression of clear cell carcinoma of the kidney. For one thing, Huaier induced human renal clear cell carcinoma cell apoptosis analyzed with flow cytometry by inhibiting the activation of the PI3K/AKT/mTOR/p70S6K/4E-BP1 pathway which is aberrantly activated in many cancers and promotes the growth and proliferation of cancer cells. The research of Xu et al. also suggests that it may inhibit renal cell carcinoma by downregulating p53 [68]. Besides, 786-O cells treated with Huaier became atrophic, irregular-shaped, and elongated and showed a special “wiredrawing” morphology; cytoplasm was vacuolated, and cell membranes became wrinkled or even dissolved, which indicated the cytotoxic effect of Huaier extract on 786-O cells and thus inhibiting the growth of solid tumors of kidney cancer. For another thing, Huaier extract affected cell motility, invasion, and migration by increasing the expression of E-cadherin, which is an important epithelial marker, and decreasing the expression of N-cadherin and vimentin which are mesenchymal markers.

Besides, Huaier extract can also act on the Hippo signaling pathway by reversing stem cell and regulates cell proliferation to play a role in the treatment of renal carcinoma. The Hippo signaling pathway consists of highly conserved kinase cascade (MAT and Lats) and downstream transcription coactivators (YAP and TAZ) and has a great effect to tissue regeneration by regulating tissue-specific stem cells. According to Malouf et al., the Hippo pathway was one of the most frequently altered pathways in sarcomatoid clear-cell renal cell carcinomas (sRCC) and YAP1 knockdown and neurofibromin 2 (NF2) reconstitution suppressed cell proliferation and tumour growth and invasion, both in vitro and in vivo [69]. The research of Guan et al. about miR-572 on proliferation and apoptosis through modulating NF2/Hippo signaling in RCC cell lines also proves the essential effect of the Hippo in renal carcinoma [70]. Huaier has a dose-dependent therapeutical effect on cancer by playing a key role to reset the tissue potential observed in such as embryonic stem cells and various progenitor cells and to restart the normal cell proliferation and specification processes [71, 72]. And this situation enables Huaier to treat renal carcinoma.

In short, Huaier reversed the EMT (epithelial-mesenchymal transition) process and inhibit the metastasis of clear renal cells, and reverse stem cell. In addition, in vivo tumourigenesis assay showed that Huaier extract could inhibit the growth of xenograft tumor in nude mice. To sum up, Huaier may serve as a promising therapeutic adjuvant for the treatment of renal cell carcinoma.

The mechanisms of HQH effects on related kidney system diseases as mentioned above are summarized in Table 1.

## 4. Conclusion

HQH is known and used in clinical medicine because of its high efficiency and low toxicity. A large number of studies have proved that HQH has an adjuvant therapeutic effect on kidney disease and even a variety of cancers. Its mechanism includes regulation of oxidative stress, immunity, autophagy, apoptosis, ferroptosis, and pyroptosis. In various kinds of kidney system-related diseases, HQH can effectively reduce hematuria and proteinuria, protect podocytes, regulate cell death, and other functions. Its medical value is worthy of further research and discovery, and it has a broad application prospect in kidney system diseases.

## Figures and Tables

**Table 1:** Mechanism of Huaiqihuang in kidney diseases.

Kidney disease	Effects	References
The mesangioproliferative glomerulonephritis (MsPGN)	Reduces the stimulation of PDGF-BB, attenuates the hyperplasia of anti-Thy-1 MsPGN, and reduces albuminuria	[62]
Primary nephrotic syndrome	Reduces the inflammatory effects of IL-18 and enhances the inflammatory inhibitory effects of IL-10 regulates the p-ERK/CHOP signaling pathway to promote podocyte proliferation and suppress podocyte apoptosis, increases the ratio of Th1/Th2, and reduces the ratio of Th17/Treg and restore balance	[5, 7, 26]
Adriamycin-induced nephropathy	Reduces proteinuria by enhancing nephrin expression and inhibit the NF-*κ*B signaling pathway, interferes with the proinflammatory and proapoptotic signaling, and promotes the nephrin and podocin protein expression	[11, 66, 73]
Renal interstitial fibrosis	Reduces infiltration of myofibroblast and downregulates the expression of *α*-SMA	[4, 66]
Allergic purpura nephritis	Downregulates the expression of TGF-*β*1 mRNA and upregulates the expression of nephrin and podocin on the septal septum	[65]
Cisplatin nephrotoxicity	Inhibits cp-induced renal tubular apoptosis and cell cycle arrest and reduces the levels of oxidative stress, inflammation and mitochondrial dysfunction	[8, 37]
IgA nephropathy	Increases the expression of nephrin and improves the anomalies of their distribution and improves the expression of CD3, CD4, and CD4/CD8	[14, 64]
Renal carcinoma	Induce human renal clear cell carcinoma cell apoptosis by inhibiting the activation of the PI3K/AKT/mTOR/p70S6K/4E-BP1 pathway and restart the normal cell proliferation on the Hippo signaling pathway	[68, 70, 71]
